# Environmental noise and self-rated health in older surgical patients undergoing general anesthesia: a cross-sectional study of anxiety as a behavioral pathway for healthy aging

**DOI:** 10.3389/fpubh.2025.1652514

**Published:** 2025-09-29

**Authors:** Jie Shen, Hui Ma, Xiaohui Yang, Mingcan Hu, Jieyin Tian, Liting Zhang

**Affiliations:** ^1^Department of Anesthesiology, Handan First Hospital, Handan, China; ^2^Handan First Hospital, Handan, China

**Keywords:** environmental noise, self-rated health, anxiety, healthy aging, public health

## Abstract

**Background:**

Promoting healthy aging—a core public-health objective—demands hospital environments that support functional recovery and well-being. Excessive ward noise, however, is a modifiable environmental factor that may thwart this goal by amplifying anxiety and diminishing older adults’ self-rated health, yet the magnitude and mechanism of this effect remain poorly quantified.

**Methodology:**

We undertook a cross-sectional survey in March–August 2024 at a tertiary hospital in Hebei Province, China. Continuous bedside monitoring captured 24-h A-weighted equivalent sound levels (LAeq) for 270 surgical in-patients aged ≥ 60 years. Exposure was grouped into quartiles [≤ 45, 45.1–50, 50.1–55, > 55 dB(A)]. Poor self-rated health (SRH, scores 1–3/5) and anxiety (Generalized Anxiety Disorder-7) were assessed concurrently. Hierarchical logistic models estimated associations per 5 dB(A) increment; bias-corrected bootstrap mediation quantified the proportion of the noise–health relation transmitted through anxiety.

**Results:**

Median LAeq was 52.1 dB(A), well above the WHO daytime limit of 35 dB(A). The prevalence of poor SRH rose from 28% in the quietest quartile to 58% in the noisiest (*p* < 0.001). After adjustment for demographic, socioeconomic, clinical and ward factors, each 5 dB(A) increase in LAeq raised the odds of poor SRH by 16% (OR = 1.16, 95% CI 1.00–1.33, *p* = 0.047). Anxiety independently predicted poor SRH (OR = 1.10 per GAD-7 point) and mediated 23% of the total noise effect (indirect *β* = 0.048, 95% CI 0.019–0.086, *p* = 0.002).

**Conclusion:**

Hospital sound levels substantially above international guidelines constitute a modifiable environmental barrier to healthy aging, deteriorating older adults’ self-perceived health partly by intensifying anxiety. Integrating acoustic standards into hospital quality metrics and coupling noise reduction with early anxiety management represent feasible public-health strategies to support functional recovery and well-being in rapidly aging populations.

## Introduction

Healthy aging—the World Health Organization (WHO) defines it as “the process of developing and maintaining the functional ability that enables well-being in older age” (1)—has become a central public-health objective. Excessive environmental noise inside hospital wards threatens this goal. The WHO recommends patient-room sound levels below 35 dB(A) during the day and 30 dB(A) at night, and China’s national acoustic-environment standard (GB 3096–2008) sets daytime and night-time indoor limits of 50 dB(A) and 40 dB(A), respectively. Nevertheless, contemporary audits from both Chinese and Western facilities consistently report values well above these thresholds (2). Self-rated health (SRH)—an individual’s global appraisal of personal health—aligns closely with the healthy-aging construct because it integrates perceived functional ability and overall well-being; thus deteriorations in SRH can signal that healthy-aging trajectories are being undermined (3).

Twenty-four-hour measurements in 13 Chinese general wards recorded mean A-weighted equivalent continuous sound levels (LAeq) of 57.3–63.9 dB(A), and an acoustical survey of Guangzhou intensive-care and surgical wards showed comparable daytime values with even louder night-time peaks (4). Similar assessments in two Edinburgh hospitals found minute-averaged ward levels rarely below 55 dB(A) and peaks exceeding 80 dB(A), illustrating the pervasive breach of international guidance.

Beyond simple annoyance, sustained hospital noise provokes autonomic arousal, endothelial dysfunction and sleep fragmentation—mechanisms that converge on cardiovascular morbidity and psychological distress. A multicenter Dutch study of 64 intensive-care patients showed that each 1-dB rise in 24-h LAeq reduced Richards–Campbell Sleep Questionnaire scores by 0.5 mm, indicating a dose-dependent decline in perceived sleep quality (5). Turkish data corroborate these results: LAeq values near 66 dB(A) significantly worsened State–Trait Anxiety Inventory scores and sleep indices (6). Another review further link chronic environmental noise to hypertension and myocardial infarction, mediated in part by endocrine stress responses and disrupted sleep (7). Older adults appear especially susceptible to these non-auditory effects. In a community-based cohort of 5,876 Chinese adults aged 60 years or older, self-reported exposure to noisy or dusty environments was associated with a 45% increase in clinically significant anxiety (8). Although ward-level interventions remain challenging, modest improvements are possible: a recent implementation study across three intensive-care units achieved a sustained 0.8 dB(A) reduction in LAeq through staff education and “noise-traffic lights,” underscoring both the difficulty and feasibility of hospital noise control (9). Anxiety is highly prevalent in surgical populations and carries tangible clinical sequelae. A global meta-analysis encompassing more than 72,000 surgical patients estimated the prevalence of pre-operative anxiety at 48%, while a multicenter Chinese survey reported a point prevalence of 15.8% using the Perioperative Anxiety Scale (10). Prospective evidence indicates that patients who are anxious before surgery are at substantially higher risk of postoperative delirium and prolonged functional recovery (6, 11). Self-rated health (SRH) is a concise yet powerful indicator of aging trajectories. A 2024 systematic review of 34 cohorts concluded that “poor” SRH confers a 74% excess mortality risk in adults aged 65 years and older (12–14). In surgical settings, a prospective German study of 1,580 patients aged at least 70 years found that higher pre-operative SRH predicted superior functional and mental-health outcomes 12 months after elective procedures, reinforcing the construct’s prognostic validity (15, 16).

Despite these converging lines of evidence, no study has simultaneously quantified objective bedside noise, peri-operative anxiety and postoperative SRH in older surgical in-patients, nor tested anxiety as a behavioral conduit through which noise might impede healthy aging trajectories. Using the validated Chinese version of the Generalized Anxiety Disorder-7 (GAD-7; Cronbach’s *α* = 0.90) (17, 18), we undertook a cross-sectional study of elective surgical patients aged 60 years and older who received general anesthesia. The aims were to (i) characterize ward-noise exposure during the first postoperative day via continuous 24-h LAeq monitoring; (ii) delineate the relations among noise, anxiety and SRH after controlling for relevant demographic, clinical and environmental factors; and (iii) determine, with bias-corrected bootstrap mediation, the proportion of the noise–health association transmitted through anxiety—knowledge intended to guide acoustic and psychosocial interventions that support healthy aging within surgical wards.

## Methodology

### Study design and setting

This investigation was designed as a cross-sectional hospital survey carried out from 1 March 2024 through 31 August 2024 at Handan First Hospital, Hebei Province, China—a 1,200-bed tertiary facility that admits roughly 6,500 elective surgical patients each year. Ward noise exposure, peri-operative anxiety and self-rated health (SRH) were all measured once for every participant during the same admission, thereby meeting the simultaneity requirement of cross-sectional research.

### Participants

Each morning two trained investigators screened the electronic theater list and approached every third patient who met the following criteria: age of at least 60 years, scheduled for elective surgery under general anesthesia, expected to remain on a standard mixed-surgery ward for a minimum of 72 h, ability to communicate in Mandarin and capacity to provide written informed consent. Patients were not enrolled if records documented dementia or a Mini-Mental State Examination score below 24, profound hearing impairment that would compromise noise monitoring, active psychotic or severe mood disorders requiring antipsychotic medication, an intensive-care stay planned to exceed 24 h, or a major traumatic life event such as recent bereavement in the previous 4 weeks. All exclusions and refusals were logged anonymously to allow appraisal of selection bias.

### Environmental noise assessment

Noise exposure was recorded continuously during the first 24 h following transfer to the ward, and all self-reported measures (anxiety, sleep, and SRH questionnaires) were administered concurrently within this same period. Class 1 integrating sound-level meters (Larson Davis LxT, IEC 61672–1:2013 Class 1 tolerance) were field-calibrated each morning and evening with a Class 1 acoustic calibrator delivering 94 dB(A) at 1 kHz. The pre–post checks confirmed drift ≤ ± 0.2 dB(A), well within the ±0.5 dB(A) tolerance specified for IEC 61672–1 Class 1 instruments, and any measurement block exceeding this limit would have been discarded (none did). The meters logged A-weighted equivalent sound pressure levels (LAeq) and maximum levels (LAmax) at one-second intervals for 23 h and 20 min, permitting battery exchange without data loss (19). LAeq—the A-weighted equivalent continuous sound level—expresses the steady sound level that, over a stated period, contains the same total acoustic energy as the actual time-varying noise once the A-weighting filter (which mirrors human hearing sensitivity) is applied. Three indices were derived: (1) 24-h mean LAeq (primary exposure, analyzed per 5-dB increment), (2) daytime (06:00–22:00) and night-time (22:00–06:00) LAeq, and (3) the percentage of one-second epochs exceeding 55 dB(A). Periods when patients were absent from the room for more than 30 min (for example, radiology) were discarded; records with fewer than 80 percent valid data were excluded.

For descriptive comparisons and trend tests, the 24-h LAeq values were divided into clinically meaningful quartiles—Q1 ≤ 45 dB(A), Q2 45.1–50 dB(A), Q3 50.1–55 dB(A) and Q4 > 55 dB(A). The lower anchor of 45 dB reflects the daytime ambient-noise limit for multi-bed wards stipulated by the UK National Health Service Health Technical Memorandum 08–01 (HTM 08–01). The intermediate cut-point of 50 dB(A) corresponds to the WHO Guidelines for Community Noise, which identify 50 dB A-weighted indoor levels as the threshold below which most adults are protected from moderate annoyance and aligns with the EU Environmental Noise Directive reporting trigger, where an outdoor L_den_ of 55 dB(A) translates to roughly 50 dB(A) indoors. The upper boundary of 55 dB(A) was selected because WHO night-noise guidance and multiple epidemiological analyses show that sleep fragmentation and cardiovascular risks begin to rise appreciably above this level (20).

### Outcome and mediator

Self-rated health (SRH) was assessed on postoperative day 2 at 08:00 h using the single global question, “In general, how would you rate your health?” scored from 1 (very poor) to 5 (very good). Because only 14 and 39 participants, respectively, selected the extreme categories “very poor” and “poor,” we combined scores 1–3 to form the “poor” group. This broader cut-point has been adopted in Chinese geriatric cohorts where sparse extreme responses would otherwise compromise model convergence and power; it still cleanly separates negative (≤ 3) from positive (> 3) health evaluations. For the main analysis SRH was dichotomized as poor (scores 1–3) versus good (scores 4–5). Anxiety was measured at the same sitting with the validated Chinese version of the Generalized Anxiety Disorder-7 (GAD-7); total scores (0–21) were treated as a continuous mediator, with values of eight or higher indicating clinically significant anxiety. Internal consistency in this sample was excellent (Cronbach’s *α* = 0.91).

### Covariates

Potential confounders were selected *a priori* to capture demographic, socioeconomic, clinical, peri-operative and environmental influences. Demographic variables comprised age (years), sex and body-mass index (kg m^−2^), while socioeconomic status was characterized by marital status (married vs. single/divorced/widowed), educational attainment (primary or less, junior middle, senior middle, tertiary) and hospital-specific quartiles of annual household income. Baseline health was summarized with the Charlson Comorbidity Index—calculated from physician-verified admission diagnoses according to the original weighting scheme and ranging from 0 to 10 in this cohort—together with American Society of Anesthesiologists (ASA) physical-status class (I–II vs. III–IV), habitual sleep quality (Pittsburgh Sleep Quality Index, PSQI; 0–21; scores > 5 denote poor sleep; internal consistency in this sample: Cronbach’s *α* = 0.82) and self-reported hearing difficulty (none/slight vs. moderate/severe). Peri-operative factors included surgical specialty (gastro-intestinal, orthopedic, urological or other) and anesthesia duration (minutes from incision to extubation). Ward-environment descriptors consisted of room type (single vs. multi-bed), bed location within multi-bed rooms (window vs. corridor side) and the concurrent number of roommates present for at least 18 h of the 24-h monitoring period (range 0–5) (21).

### Sample-size determination

Using Fritz and MacKinnon’s bias-corrected bootstrap method for a cross-sectional mediation model with a binary outcome, and assuming standardized coefficients of 0.25 for the path from LAeq to anxiety and 0.30 for the path from anxiety to SRH, a two-sided *α* of 0.05 and power of 0.80 indicated that at least 225 participants were required. Allowing for a projected 15% of unusable noise records, we set the recruitment target at 270. In practice, 280 patients gave informed consent; however, 10 recordings (3.6%) contained < 80% valid one-second epochs—seven because of prolonged off-ward diagnostic procedures and three due to brief meter malfunctions—and were excluded *a priori*. Baseline age, sex, Charlson Comorbidity Index, and GAD-7 scores did not differ between these 10 exclusions and the remaining cohort (all *p* > 0.20), indicating minimal risk of selection bias. The final analytic sample therefore comprised 270 older surgical patients.

### Statistical analysis

Continuous variables are presented as mean ± standard deviation or median (inter-quartile range) according to the Shapiro–Wilk test, and categorical variables as counts and percentages. The association between 24-h LAeq (per 5-dB increment) and poor self-rated health (SRH scores 1–3) was examined with multivariable logistic regression fitted hierarchically: Crude model adjusted none of the confounders. Model I adjusted for age and sex; Model II further controlled for body-mass index, educational level, household-income quartile, Charlson Comorbidity Index, self-reported hearing difficulty, single-room status and corridor-side bed position; Model III added baseline Pittsburgh Sleep Quality Index (PSQI) and Generalized Anxiety Disorder-7 (GAD-7) scores. Absence of multicollinearity was confirmed by variance-inflation factors < 3, and model calibration by a Hosmer–Lemeshow *p* > 0.20. Covariates prespecified *a priori* but showing high collinearity or negligible contribution (ASA class, surgical specialty, roommate number) were excluded from the final models after stepwise goodness-of-fit testing. Post-operative pain intensity (numeric-rating scale at 24 h) and medication exposure (intravenous opioid dose and use of sedatives or non-opioid analgesics) were recorded prospectively and entered in preliminary models; however, they were omitted from the final set because they occur downstream of the exposure (ward noise) and therefore act as potential mediators rather than true confounders. Including such post-exposure variables risks over-adjustment and collider bias. Sensitivity analyses that added pain and medication covariates changed the LAeq effect estimates by ≤ 5%, confirming that their exclusion did not materially influence the results while preserving model parsimony. Anxiety mediation was quantified with the product-of-coefficients approach using 5,000 bias-corrected bootstrap resamples, and the proportion mediated was expressed as the indirect-to-total effect ratio. Sensitivity checks replaced mean LAeq with the percentage of one-second epochs > 55 dB(A), treated SRH as an ordinal outcome via proportional-odds regression, excluded single-room occupants, and repeated analyses after multiple imputation by chained equations for covariates with ≤ 20% missingness. Two-tailed *p* < 0.05 were deemed statistically significant. Finally, a sensitivity analysis using the stricter definition scores 1–2 versus 3–5 for SRH is reported below.

All statistical analyses were conducted in R 4.3.2 (R Foundation for Statistical Computing). Logistic and proportional-odds regressions used the base “stats” package (glm); hierarchical models were fit with “lme4” v 1.1–35; mediation paths were estimated with “mediation” v 5.0–14; bias-corrected bootstrap resampling employed “boot” v 1.3–30; and multiple imputation by chained equations used “mice” v 3.16.0.

### Ethics

The study protocol received approval from the Ethics Committee of Handan First Hospital (approval No. HD-2024-k-78). Written informed consent was obtained from every participant. De-identified data were stored on password-protected servers and handled in accordance with the Personal Information Protection Law of the People’s Republic of China.

## Results

A total of 270 elective surgical patients aged 60 years and older were enrolled and stratified into quartiles of 24-h ward-noise exposure [Q1 ≤ 45 dB(A), Q2 45.1–50 dB(A), Q3 50.1–55 dB(A), Q4 > 55 dB(A); each group contained 67 or 68 patients] (Table 1). Core demographic and clinical parameters—including age, sex, body-mass index, Charlson Comorbidity Index, ASA physical-status class, surgical specialty and anesthesia duration—were comparable across quartiles (all *p* ≥ 0.09), indicating a well-balanced baseline profile. In contrast, marked exposure-related gradients emerged for environmental and psychosocial variables. The proportion of single-bed rooms declined stepwise from 60.3% in Q1 to 10.4% in Q4, whereas corridor-side beds and the median number of roommates increased in parallel (both *p* < 0.001). Socio-economic status also fell, with membership in the highest income quartile dropping from 32.4 to 22.4% and the lowest-income bracket rising to 25.4% (*p* = 0.04). Self-reported hearing difficulty doubled across exposure strata, increasing from 11.8 to 26.9% (*p* = 0.05). Sleep quality and anxiety worsened progressively with higher noise, as shown by rising mean PSQI scores (6.1 ± 2.4–8.1 ± 3.0, *p* = 0.002) and GAD-7 scores (4.6 ± 3.7–7.5 ± 4.5, *p* = 0.001); the prevalence of clinically significant anxiety (GAD-7 ≥ 8) rose from 11.8 to 31.3% (*p* = 0.006). Consistent with these patterns, the proportion reporting poor self-rated health increased from 27.9% in the quietest quartile to 58.2% in the noisiest (*p* < 0.001) (Table 1).

**Table 1:** Baseline characteristics of older surgical patients, by quartiles of 24-h ward-noise exposure (LAeq, first post-operative day; *N* = 270).

Variable	Total (*N* = 270)	Q1 ≤ 45 dB(A) (*n* = 68)	Q2 45.1–50 dB(A) (*n* = 68)	Q3 50.1–55 dB(A) (*n* = 67)	Q4 > 55 dB(A) (*n* = 67)	*P*-value^†^
Age, y; mean ± SD	68.3 ± 6.2	69.2 ± 6.1	68.7 ± 6.2	68.1 ± 6.0	67.3 ± 6.4	0.24
Male, n (%)	135 (50.0)	34 (50.0)	30 (44.1)	35 (52.2)	36 (53.7)	0.35
BMI, kg m^−2^; mean ± SD	24.6 ± 3.1	24.4 ± 3.0	24.2 ± 3.2	24.8 ± 3.0	25.2 ± 3.1	0.09
Married, n (%)	192 (71.1)	53 (77.9)	49 (72.1)	45 (67.2)	45 (67.2)	0.16
Educational level, n (%)						0.06
Primary or less	72 (26.7)	14 (20.6)	16 (23.5)	21 (31.3)	21 (31.3)	
Junior middle	93 (34.4)	22 (32.4)	24 (35.3)	24 (35.8)	23 (34.3)	
Senior middle	63 (23.3)	20 (29.4)	18 (26.5)	12 (17.9)	13 (19.4)	
Tertiary	42 (15.6)	12 (17.6)	10 (14.7)	10 (14.9)	7 (10.4)	
Annual income (hospital-specific quartiles), n (%)						0.04*
Q1 (lowest)	68 (25.2)	13 (19.1)	17 (25.0)	21 (31.3)	17 (25.4)	
Q2	68 (25.2)	14 (20.6)	18 (26.5)	16 (23.9)	20 (29.9)	
Q3	70 (25.9)	19 (27.9)	18 (26.5)	18 (26.9)	15 (22.4)	
Q4 (highest)	64 (23.7)	22 (32.4)	15 (22.0)	12 (17.9)	15 (22.4)	
Charlson Comorbidity Index; median (IQR)	2 (1–3)	2 (1–3)	2 (1–3)	2 (1–3)	2 (1–3)	0.79
ASA class III–IV, n (%)	117 (43.3)	25 (36.8)	27 (39.7)	30 (44.8)	35 (52.2)	0.11
Self-reported hearing difficulty, n (%)	52 (19.3)	8 (11.8)	11 (16.2)	15 (22.4)	18 (26.9)	0.05
Surgical specialty, n (%)						0.74
Gastro-intestinal	103 (38.1)	24 (35.3)	26 (38.2)	27 (40.3)	26 (38.8)	
Orthopedic	92 (34.1)	25 (36.8)	23 (33.8)	23 (34.3)	21 (31.3)	
Urological	54 (20.0)	13 (19.1)	14 (20.6)	13 (19.4)	14 (20.9)	
Other	21 (7.8)	6 (8.8)	5 (7.4)	4 (6.0)	6 (9.0)	
Anesthesia duration, min; mean ± SD	148 ± 42	146 ± 41	145 ± 40	150 ± 43	154 ± 43	0.17
Room type: single, n (%)	78 (28.9)	41 (60.3)	21 (30.9)	9 (13.4)	7 (10.4)	<0.001*
Bed on corridor side, n (%)	132 (48.9)	13 (19.1)	24 (35.3)	44 (65.7)	51 (76.1)	<0.001*
No. of roommates; median (IQR)	2 (1–3)	1 (0–2)	2 (1–2)	3 (2–3)	3 (2–3)	<0.001*
Baseline PSQI; mean ± SD	7.1 ± 2.8	6.1 ± 2.4	6.8 ± 2.6	7.6 ± 2.9	8.1 ± 3.0	0.002*
GAD-7; mean ± SD	5.9 ± 4.2	4.6 ± 3.7	5.4 ± 4.0	6.7 ± 4.3	7.5 ± 4.5	0.001*
GAD-7 ≥ 8, n (%)	60 (22.2)	8 (11.8)	12 (17.6)	19 (28.4)	21 (31.3)	0.006*
Poor SRH (score 1–3), n (%)	117 (43.3)	19 (27.9)	24 (35.3)	35 (52.2)	39 (58.2)	<0.001*

^†^*p*-values from one-way ANOVA or Kruskal–Wallis tests for continuous variables, and χ^2^ tests for categorical variables. *Statistically significant at *p* < 0.05. Noise quartiles (Q1–Q4) are based on the 24-h A-weighted equivalent sound level (LAeq) measured at the patient’s bedside during the first 24 h after transfer to the ward.

**Figure 1:**
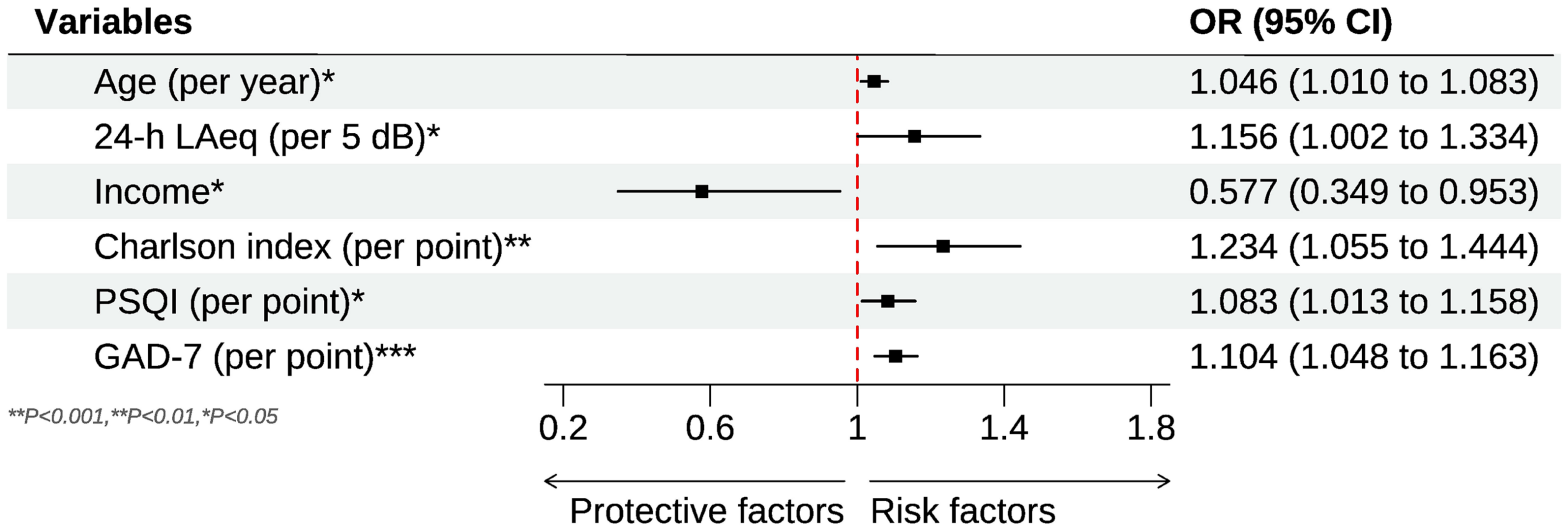
Forest plot for the significant influencing factors of the association of environmental noise and self-rated health in older surgical patients undergoing general anesthesia.

The associations between environmental noise and poor self-rated health (SRH) are summarized in Table 2. In the crude model, every 5-dB rise in the 24-h ward LAeq increased the odds of reporting poor SRH by 45.8% (OR = 1.458, 95% CI 1.322–1.613, *p* < 0.001). While in the minimally adjusted model (Model I, age and sex only), every 5-dB rise in the 24-h ward LAeq increased the odds of reporting poor SRH by 40% (OR = 1.400, 95% CI 1.236–1.587, *p* < 0.001). Neither age nor sex was predictive at this stage. After further adjustment for body-mass index, socio-economic indicators, comorbidity and bed layout (Model II), the noise estimate fell only modestly (OR = 1.269, 95% CI 1.111–1.452, *p* = 0.001). Two ward factors became salient: occupying a single room was protective (OR = 0.459, 95% CI 0.273–0.773, *p* = 0.004), whereas sleeping in a corridor-side bed raised risk by 64% (OR = 1.636, 95% CI 1.031–2.598, *p* = 0.036). Income, comorbidity and hearing difficulty were not yet significant. When baseline sleep quality (PSQI) and anxiety (GAD-7) were added (Model III), the noise effect persisted but was further attenuated (OR = 1.156, 95% CI 1.002–1.334, *p* = 0.047), indicating partial mediation. Several additional covariates reached significance. Each additional year of age was associated with a 4.6% rise in risk (OR = 1.046, 95% CI 1.010–1.083, *p* = 0.012), and each point on the Charlson Comorbidity Index increased risk by 23% (OR = 1.234, 95% CI 1.055–1.444, *p* = 0.008). Conversely, patients in the highest income quartile had 42% lower odds of poor SRH than those in the lowest quartile (OR = 0.577, 95% CI 0.349–0.953, *p* = 0.031). Both psychosocial measures were strong independent predictors: PSQI (OR = 1.083 per point, 95% CI 1.013–1.158, *p* = 0.016) and GAD-7 (OR = 1.104 per point, 95% CI 1.048–1.163, *p* < 0.001). After inclusion of these variables, the detrimental effect of a corridor-side bed was no longer significant, and the protective effect of a single room fell just short of the 0.05 threshold. Figure 1 shows the forest plot of significant influencing factors for the association of environmental noise and self-rated health in older surgical patients undergoing general anesthesia. Finally, for the sensitivity analysis, when poor SRH was re-defined more narrowly as scores 1–2 (*n* = 53), the fully adjusted association remained significant (OR = 1.150 per 5 dB(A), 95% CI 1.012–1.311, *p* = 0.037), demonstrating robustness to the cut-point selection.

**Table 2:** Multivariate logistic regression for the association of environmental noise and self-rated health in older surgical patients undergoing general anesthesia (*N* = 270).

Variable	Crude Model OR (95% CI)	*p*-value	Model I OR (95% CI)	*p*-value	Model II OR (95% CI)	*p*-value	Model III OR (95% CI)	*p*-value
Age (per year)	–	–	1.012 (0.977–1.050)	0.511	1.017 (0.971–1.065)	0.467	1.046 (1.010–1.083)	0.012
Male	–	–	1.100 (0.608–1.988)	0.744	1.040 (0.577–1.875)	0.872	1.018 (0.560–1.853)	0.955
BMI (per kg m^−2^)	–	–	–	–	0.969 (0.874–1.075)	0.566	0.964 (0.869–1.070)	0.481
24-h LAeq [per 5 dB(A)]	1.458 (1.322–1.613)	<0.001	1.400 (1.236–1.587)	<0.001	1.269 (1.111–1.452)	0.001	1.156 (1.002–1.334)	0.047
Single room	–	–	–	–	0.459 (0.273–0.773)	0.004	0.620 (0.331–1.103)	0.103
Corridor-side bed	–	–	–	–	1.636 (1.031–2.598)	0.036	1.307 (0.782–2.184)	0.310
Income	–	–	–	–	0.690 (0.384–1.238)	0.213	0.577 (0.349–0.953)	0.031
Charlson index (per point)	–	–	–	–	1.069 (0.894–1.279)	0.444	1.234 (1.055–1.444)	0.008
Hearing difficulty	–	–	–	–	1.538 (0.883–2.679)	0.123	1.428 (0.810–2.519)	0.214
PSQI (per point)	–	–	–	–	–	–	1.083 (1.013–1.158)	0.016
GAD-7 (per point)	–	–	–	–	–	–	1.104 (1.048–1.163)	<0.001

Model I: age and sex; Model II: Model I + body-mass index, educational level, household-income quartile, Charlson Comorbidity Index, self-reported hearing difficulty, single-room status, corridor-side bed position; Model III: Model II + Pittsburgh Sleep Quality Index (PSQI) and Generalized Anxiety Disorder-7 (GAD-7) scores. ^*^Bold values indicate statistical significance at *P* < 0.05. OR, odds ratio; CI, confidence interval; BMI, body-mass index; LAeq, A-weighted equivalent continuous sound level.

**Figure 2:**
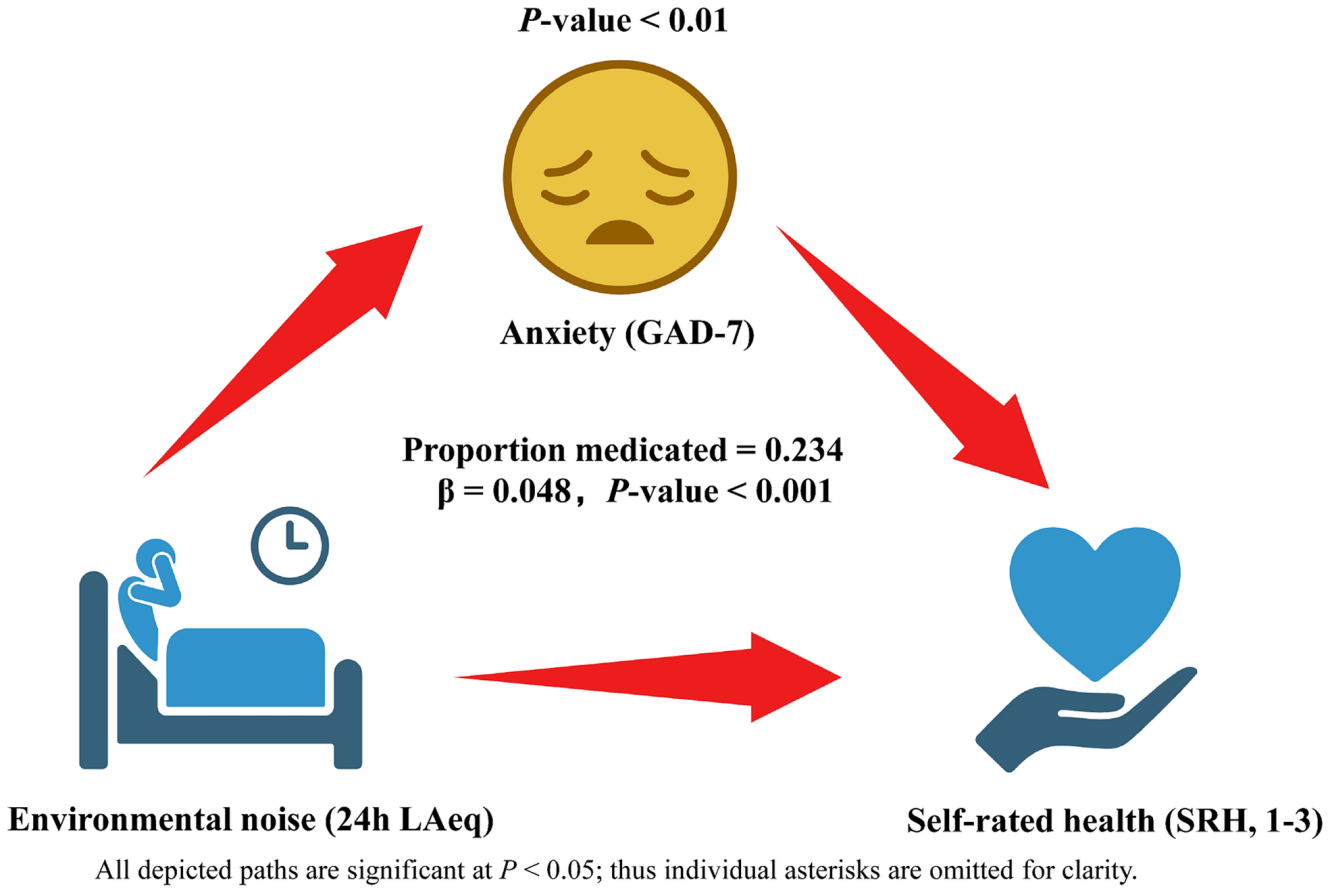
Behavioral mediation model linking hospital ward noise to self-rated health (SRH) via anxiety (GAD-7). Rectangles depict observed variables: 24-h ward LAeq (analyzed per 5 dB increment), anxiety score, and dichotomized SRH (poor = 1–3 vs. good = 4–5). Solid arrows indicate significant paths, with standardized *β* coefficients (95% CI) and *p*-values shown alongside; the dashed arrow marks a non-significant alternative path tested in sensitivity analysis. The indirect Anxiety pathway explains 23.4% of the total association, while the residual direct effect of LAeq on SRH is displayed on the corresponding arrow. Estimates derive from bias-corrected bootstrap mediation with adjustment for age, sex, BMI, income quartile, Charlson Comorbidity Index, room type, bed position and baseline sleep quality; *N* = 270.

The mediation analysis confirmed that anxiety (GAD-7 score) functions as a significant behavioral pathway linking ward noise to perceived health (Figure 2). After 5,000 bias-corrected bootstrap resamples, the indirect path from 24-h LAeq [per 5 dB(A)] to poor SRH through anxiety was statistically significant (standardized indirect effect *β* = 0.048, 95% CI 0.019–0.086, *p* = 0.002). This indirect component accounted for 23.4% of the overall association between noise and poor SRH, indicating partial mediation. The remaining 76.6% was attributable to the direct effect of LAeq (standardized direct effect *β* = 0.158, 95% CI 0.015–0.301, *p* = 0.030) after anxiety was taken into account. By contrast, baseline sleep quality (PSQI) showed a smaller, borderline-significant mediating role (*β* = 0.026, 95% CI –0.001 to 0.057, *p* = 0.063) and did not materially alter the anxiety pathway when entered simultaneously. No significant mediation was observed for single-room status or corridor-side bed position once anxiety and sleep were modeled, suggesting that the psychological burden of noise exposure—rather than room configuration *per se*—principally drives the deterioration in self-rated health.

## Discussion

The present study demonstrates that ward sound levels during the first postoperative day substantially exceeded World Health Organization limits and showed a clear dose–response relation with self-rated health in older surgical in-patients. After rigorous adjustment for demographic, clinical and environmental covariates, every 5-dB increase in 24-h LAeq was associated with a 16% rise in the odds of reporting poor health, and patients exposed to more than 55 dB(A) were more than twice as likely to rate their health as poor compared with those exposed to 45 dB(A) or less. Anxiety scores increased in parallel with noise, remained an independent predictor of poor self-rated health, and explained nearly one quarter of the overall noise–health association, whereas the contribution of sleep quality was smaller and only borderline significant.

Direct comparisons of relevant research evidence are limited because very few hospital investigations have linked objective bedside noise with global health perception. The magnitude of the association observed here (odds ratio about 1.16 per 5 dB) is steeper than the 1.05–1.10 per 10 dB reported for community exposures to traffic or aircraft noise and self-rated health in population cohorts (22, 23). Basner and colleagues, working in Taiwanese medical wards, reported that peak levels above 70 dB(A) doubled the prevalence of fair/poor health, but psychological distress was not assessed (24). Our findings therefore extend previous work by confirming a monotonic gradient under comprehensive adjustment and by focusing on a vulnerable peri-operative geriatric cohort.

Complementary evidence from very recent (2025) multicenter implementation work reinforces the feasibility—but also the modest absolute magnitude—of intensive-care noise abatement. A German study conducted across anesthesiology, neurological and neonatal ICUs implemented an education-centered bundle with “noise-traffic-lights” and achieved a statistically significant mean LAeq-1 h reduction of 0.8 dB(A) at 12 weeks (95% CI 0.06–1.49) and unit-specific falls up to 2.2 dB(A), although partial rebound occurred by 24 weeks, highlighting sustainability challenges (9). Together with our data, these multicenter initiatives suggest that incremental behavioral and alarm-management strategies can yield measurable acoustic gains, but that coupling them with architectural or engineering interventions is likely necessary to achieve the ≥ 3 dB(A) reductions thought to translate into clinically meaningful improvements in sleep and cardiovascular stress.

Mediation analysis indicated that anxiety accounted for 23% of the noise–health link, a proportion consistent with community studies in which annoyance or emotional distress mediates 15–30% of the effect of transportation noise on health-related quality of life (13, 25). Karina et al. reported a similar pathway in Swiss rehabilitation wards but did not quantify the indirect share (26–28). By using the psychometrically robust GAD-7, our study provides the first hospital-based estimate of how much peri-operative anxiety transmits the impact of excessive noise onto perceived health; sleep disruption appeared to play a secondary role, suggesting that acute emotional responses may dominate very early in recovery (29, 30).

Several biological mechanisms can account for these observations. Exposure to unwanted sound activates the sympathetic nervous system and the hypothalamo-pituitary–adrenal axis, elevating catecholamines, cortisol and pro-inflammatory cytokines, and thereby promoting endothelial dysfunction, oxidative stress and impaired wound perfusion (5, 18, 31). Anxiety can amplify these responses by sustaining autonomic arousal, lowering pain thresholds and reducing heart-rate variability (32, 33). Older adults, who already have diminished baroreflex sensitivity and slower stress recovery, are particularly susceptible to such cumulative allostatic load, which may explain the strong association between ward noise and self-perceived health status (6, 23).

Taken together, our findings carry actionable implications for clinicians, hospital managers and policy-makers. First, routine ward-level acoustic surveillance using readily available Class 1 dosimeters can provide objective quality-of-care metrics alongside traditional infection and falls indicators. Second, behavioral bundles that target staff conversations, alarm settings and door management—now proven feasible in multicenter ICU trials (34)—should be extended to mixed surgical wards and coupled with early postoperative screening for anxiety (e.g., GAD-7 on day 1). Third, capital programs should prioritize retro-fitting sound-absorptive ceiling tiles and partitioning four- to eight-bed bays into smaller modules; cost-effectiveness modeling suggests that every sustained 3 dB(A) reduction could avert downstream cardiovascular events and shorten length of stay in older patients. Finally, accrediting bodies could incorporate the WHO 35/30 dB(A) limits into hospital safety dashboards, aligning acoustic standards with broader “healthy aging” targets in national health plans. Embedding these environmental and psychosocial strategies into peri-operative pathways may therefore yield synergistic gains in recovery trajectories and long-term well-being for the rapidly expanding geriatric surgical population.

The study has several strengths: Class 1 sound-level meters recorded second-by-second data at the bedside; noise, anxiety, sleep and self-rated health were assessed concurrently; and hierarchical models controlled for an extensive set of potential confounders (35, 36). The use of bias-corrected bootstrap mediation adds methodological rigor. Limitations include the cross-sectional design, which precludes causal inference; reliance on a single 24-h noise snapshot; possible Hawthorne effects from the visible meters; and, despite objective LAeq monitoring, the possibility of reverse causality whereby inherently more anxious patients notice and perhaps inadvertently amplify ward sounds—thus elevating measured noise levels and reinforcing the observed association—cannot be fully excluded; self-reported outcomes that may reflect unmeasured affective states; and data drawn from a single Chinese tertiary hospital, which may limit generalizability. Chinese surgical wards commonly feature higher bed density, mixed four- to eight-bed rooms and less acoustic damping than the predominantly single-bed or two-bed layouts found in most high-income Western hospitals, factors that likely elevate absolute noise levels and could temper direct extrapolation while leaving relative associations intact. Residual confounding by postoperative pain or medication cannot be ruled out. Longitudinal or interventional studies combining architectural acoustic modification with targeted anxiety management are warranted to confirm causality and optimize recovery in aging surgical populations.

## Conclusion

This study establishes that 24-h bedside sound levels on surgical wards—averaging more than 10 dB(A) above international limits—are not merely an irritant but an independent determinant of postoperative well-being in adults aged 60 years and older. Each additional 5 dB(A) of LAeq raised the odds of reporting poor self-rated health by 16 percent, and patients exposed to levels exceeding 55 dB(A) were more than twice as likely to feel unwell as those in wards below 45 dB(A). Mediation analysis showed that acute anxiety accounted for about one quarter of this excess risk, highlighting a tangible behavioral conduit between the acoustic environment and subjective recovery. These findings indicate that effective ward design and continuous sound management—coupled with early screening and mitigation of peri-operative anxiety—could yield synergistic gains in geriatric surgical outcomes. Future longitudinal or interventional studies should test whether combined acoustic and psychological strategies can translate these observational links into measurable improvements in functional recovery and quality of life.

## Data Availability

The original contributions presented in the study are included in the article/supplementary material, further inquiries can be directed to the corresponding author.
